# U.S. public opinion about the safety of gene editing in the agriculture and medical fields and the amount of evidence needed to improve opinions

**DOI:** 10.3389/fbioe.2024.1340398

**Published:** 2024-02-16

**Authors:** Brandon R. McFadden, Joy N. Rumble, Kathryn A. Stofer, Kevin M. Folta

**Affiliations:** ^1^ Department of Agricultural Economics and Agribusiness, University of Arkansas, Fayetteville, AR, United States; ^2^ Department of Agricultural Communication, Education, and Leadership, The Ohio State University, Columbus, OH, United States; ^3^ Department of Agricultural Education and Communication, University of Florida, Gainesville, FL, United States; ^4^ Horticultural Sciences Department, University of Florida, Gainesville, FL, United States

**Keywords:** gene editing, biotechnology, public opinion, consumer acceptance, science communication

## Abstract

**Introduction:** Implementation of gene editing in agriculture and medicine hinges on public acceptance. The objectives of this study were to explore U.S. public opinion about gene editing in agricultural and medical fields and to provide more insight into the relationship between opinions about the safety of gene editing and the potential impact of evidence to improve opinions about safety.

**Methods:** Data were from two samples of U.S. respondents: 1,442 respondents in 2021 and 3,125 respondents in 2022. Survey respondents provided their opinions about the safety of gene editing in the agricultural and medical fields and answered questions about the number of studies or length of time without a negative outcome to improve opinions about the safety of gene editing in the agricultural and medical fields.

**Results:** Results indicated that respondents in both samples were more familiar, more likely to have an opinion about safety, and more positive about the safety of gene editing in the agricultural field than in the medical field. Also, familiarity was more closely associated with opinions about safety than the strength of opinions.

**Discussion:** These findings add to the literature examining perceptions of gene editing in the agricultural or medical fields separately. Opinions about the safety of gene editing were generally more favorable for respondents who were aware of the use of gene editing. These results support a proactive approach for effective communication strategies to inform the public about the use of gene editing in the agricultural and medical fields.

## 1 Introduction

Gene editing, the process of precisely changing or deleting a few “letters” of DNA, has already contributed to agricultural and medical advancement, with many more applications in development. However, public perception may hinder implementation and it is unclear what U.S. public opinion about safety may vary across the two fields. It has been argued that the lack of proactive public dialogue surrounding the primary introduction of genetically modified organisms (GMOs) “did irreparable damage to the emerging scientific field of genetic engineering,” and that the continued expansion of gene editing in the agricultural and medical fields has led many to call for “broad public dialogue” about the technology (NASEM, 2017). These calls are backed by the desire to “avoid unjustifiably inhibiting innovation, stigmatizing new technologies, or creating trade barriers” (Holdren et al., 2019). At the same time, news reports bring attention and fear to medical uses that can cause the public to question the ethical use, but also present opportunities for conversation about benefits and risks (Zhang et al., 2021). As technologies advance, it is vital to understand and engage the public in conversations about gene editing in agricultural and medical contexts. Opinions about the safety of gene editing in one field may provide the public with context for use in another field. It is, therefore, critical to assess public sentiment and barriers to acceptance.

Public aversion to the use of related biotechnology in agriculture has been well-documented (Lusk et al., 2005), despite support from the scientific community. For example, a 2014 Pew Research survey of U.S. adults and researchers affiliated with the American Association for the Advancement of Science (AAAS) estimated that 88% of AAAS members agreed that *genetically modified* foods were safe to consume compared to only 37% of adults (Funk et al., 2015). It is reasonable to posit that gaps between the opinions of researchers and the public are due to a lack of public understanding of evidence showing that approved biotechnology applications are safe. The public is likely unaware that more than 4,000 science-based risk assessments have concluded genetically engineered crops do not pose greater risks than conventionally bred crops (ISAAA, 2019), or that the National Academies of Sciences, Engineering, and Medicine concluded there had not been any scientifically documented human safety issues after 30 years of evaluation (NASEM, 2016).

Recent research on public opinion toward the use of biotechnology in agriculture has focused on differences in opinions between the use of gene editing and traditional genetic modification (transgenesis). These studies concluded that the public generally supports gene editing in agriculture more than transgenics (Kato-Nitta et al., 2019; Yang and Hobbs, 2020). However, public acceptance of gene editing compared to transgenic technology may differ due to some familiarity with gene editing for medical purposes. When participants in U.S. focus groups were asked what they thought about when hearing the words gene editing, the medical field was discussed more frequently and extensively than agriculture (McFadden et al., 2021a). The announcement of gene-edited twins in China increased public awareness of medical applications, as there was a surge in online searches for gene editing following the announcement (McFadden et al., 2021b). Yet, it is not clear that U.S. adults see a strong connection between gene editing in agricultural and medical fields (Watanabe et al., 2020), nor do we understand how their thoughts may vary across potential uses within fields. In Australia, survey respondents supported the use of gene editing in the agricultural and medical fields for research purposes; however, respondents were more supportive of gene-editing humans to improve health than animals used for food (Critchley et al., 2019).

Other recent research has examined differences in acceptance across agricultural commodities gene-edited for disease resistance (animal vs. plant) and acceptance for gene editing a host or vector (tree vs. insect) to reduce disease pressure (McFadden et al., 2021a; Bush et al., 2022). Respondents were more accepting of gene-editing plants than trees (Bush et al., 2022), and there were similar acceptance levels for trees and insects (McFadden et al., 2021b). Much research examining public attitudes about gene editing in the medical field has focused on which medical changes are acceptable to the public and the demographic characteristics associated with opinions. In general, results from public opinion research indicate support for therapeutic uses of gene-edited and aversion for non-disease uses that are cosmetic or otherwise alter physical characteristics (Gaskell et al., 2017; Treleaven and Tuch, 2018; Critchley et al., 2019; McCaughey et al., 2019; Jedwab et al., 2020; Watanabe et al., 2020; McFadden et al., 2021a; Kobayashi et al., 2022).

Calls for “more research” are made so often that some journals have banned using the phrase (Godlee, 2010). Science engagement must begin asking “how much?” or “what kinds?” of research are needed and or whether a different approach to promoting acceptance is required (Hering, 2016). Science communication promoting technology diffusion is more complex than simply reducing knowledge gaps (Simis et al., 2016), and behavioral responses to evidence can cause rejection of evidence that conflicts with prior beliefs about the safety of genetic engineering (McFadden and Lusk, 2015). Further, affective reactions like disgust can induce absolute moral opposition that devalues any evidence about the benefits of genetic engineering (Scott et al., 2016). Conversely, experience and familiarity with topics can lead to more favorable opinions (Liu and Priest, 2009).

The objectives of this study were to explore U.S. public opinion about gene-editing in the agricultural and medical fields and to provide more insight into the relationship between opinions about the safety of gene editing and the potential impact of evidence to improve opinions about safety. To complete the objectives of this study, data were collected from an online survey distributed to two samples of respondents. Survey respondents provided answers to five questions used as variables in this study. Asked in the following order, the questions measured familiarity with gene editing, the strength of opinion about the safety of gene editing, opinions about the safety of gene editing, and two questions measuring the amount of evidence needed to improve an opinion about the safety of gene editing (i.e., number of studies and amount of time without a negative outcome). Survey respondents answered these questions for gene editing in the agricultural and medical fields separately, with the order of presentation randomized across respondents.

## 2 Materials and methods

Data were collected at two time periods using surveys distributed by Qualtrics to online samples of U.S. adult respondents. The Institutional Review Board at the University of Delaware approved both surveys (IRB No. 1351707-4 and IRB No. 1351707-5). Collecting data from two samples allowed us to examine the stability of results across groups of respondents and time. The first survey was fielded from 3rd February to 1st March of 2021, and data were collected from 1,442 respondents. The second survey was fielded from 16th June to 25th July of 2022, and data were collected from 3,125 respondents. A quota-based sampling approach was used to obtain samples representative of the U.S. population across respondent characteristics of age, gender, income, and education. Images of the survey questions asked for the characteristics of respondents are shown in Supplementary Figure S1, and the summary statistics for the characteristics in samples 1 and 2 are presented in Supplementary Table S1.

### 2.1 Survey questions

Survey respondents answered questions about gene editing in the agricultural and medical fields. Questions for gene editing in the agricultural or medical fields were presented in individual blocks so that questions for the different fields were asked separately. Also, the order in which the question blocks were presented was randomized across respondents to minimize possible order effects associated with sequentially responding to questions about the two fields. Images of the survey questions asked about gene editing in the agriculture field are shown in Supplementary Figure S2 and questions asked about gene editing in the medical field are shown in Supplementary Figure S3.

Respondents were asked if they had heard or read about the use of gene editing in the context of “food and agriculture” or “health and medical” to determine awareness before asking if they had an opinion about the safety of gene editing; response options for awareness were a) No, b) Yes, I have heard or read a little, and c) Yes, I have heard or read a lot. Next, respondents were asked if they had an opinion about the safety of gene editing; response options were a) No, b) Yes, I have a weak opinion, and c) Yes, I have a strong opinion. Then, opinions about the safety of gene editing were collected using a five-point response scale (extremely unsafe, somewhat unsafe, neither safe nor unsafe, somewhat safe, extremely safe). For data analysis, these responses were collapsed into three categories (extremely/somewhat unsafe, neither safe nor unsafe, and somewhat/extremely safe).

The following two questions were then asked to examine the amount of evidence needed to change opinions about the safety of gene editing. One question asked what amount of research concluding that gene editing was safe was needed to improve opinions about safety; response options varied by the number of studies and were a) 1–25 studies, b) 26–50 studies, c) 51–75 studies, d) 76–100 studies, e) 100+ studies, and f) No amount of research will improve my opinion. There are often calls for “more research” by the public audiences; however, it is unclear how much is enough. The other question asked about the amount of time without a negative outcome was needed to improve opinions about safety; response options varied by the number of years and were a) 1–3 years, b) 4–6 years, c) 7–9 years, d) 10–20 years, e) 20+ years, and f) No amount of time without a negative outcome will improve opinion.

### 2.2 Statistical analysis

Within-sample heterogeneity in responses to questions about gene editing in the agricultural and medical fields was estimated using Chi-square tests of independence. These tests determined whether familiarity, opinion strength, opinion about safety, or the evidence needed to improve opinion varied across gene-edited in the agricultural and medical fields.

Chi-square tests of independence were also estimated within the agricultural and medical fields to determine relationships between familiarity and opinion strength, relationships between opinion strength and opinions about safety, and relationships between opinions about safety and the amount of evidence needed to improve opinions about safety.

To determine the effects of familiarity and opinion strength on opinions about safety, ordered logistic regression models were estimated for both samples’ responses to the agricultural and medical fields. Ordered logistic regression models were selected because the dependent variable, opinion about safety, was categorical and took the value of 0 for extremely/somewhat unsafe, 1 for neither safe nor unsafe, and 2 for somewhat/extremely safe. Indicator variables were created for levels of familiarity and opinion strength, which were used as independent variables in estimation. For familiarity, indicator variables were created for respondents who selected “Yes, I have heard or read a little” or “Yes, I have heard or read a lot” (the “No” responses were used as the base). For opinion strength, indicator variables were created for respondents who selected “Yes, I have a weak opinion” or “Yes, I have a strong opinion” (the “No” responses were used as the base).

Ordered logistic regression models were also estimated to determine the effects of familiarity, opinion strength, and opinions about safety on the evidence needed to improve opinions about safety. The dependent variables for the amount of research and the amount of time necessary to improve opinions were categorical and increased with the amount of evidence required. The same independent variables used for familiarity and opinion strength in the ordered logistic regression models previously described were also used for these estimations, and independent variables were added for opinions about safety. For opinions about safety, indicator variables were created for respondents who selected “neither safe nor unsafe” or “somewhat/extremely safe” (the “extremely/somewhat unsafe” responses were used as the base).

## 3 Results

Frequency distributions for responses to the five questions used as variables in this study are presented in Supplementary Table S2. Also presented in Supplementary Table S2 are results from Wilcoxon matched-pairs signed-rank tests that were estimated to examine heterogeneity in responses between agricultural and medical gene-edited fields within a sample. Respondents in both samples reported they were more familiar with and more likely to have a stronger opinion about gene editing in the agricultural field. Nearly 66% of sample 1 and 75% of sample 2 had some familiarity with gene editing in agriculture, compared to about 61% and 63% in samples 1 and 2 for medical. About 62% of sample 1 and 66% of sample 2 had an opinion about the safety of gene editing in agriculture, while about 56% and 58% of samples 1 and 2 had formed opinions in the medical field. However, opinions about the safety of gene editing were similar across agricultural and medical fields, with around 40%–44% of respondents stating that gene editing in the two fields was somewhat or extremely safe.

The correlations between question responses are shown in Supplementary Table S3 to provide an understanding of linear relationships between the variables. Linear relationships were generally weak. However, there were moderate correlations between familiarity and strength of opinion, ranging from 0.56 to 0.70, and between the two variables exploring the amount of evidence necessary to improve opinions about safety, ranging from 0.58 to 0.66.

**FIGURE 1:**
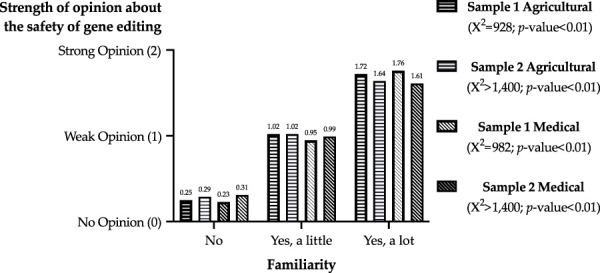
Strength of opinion about the safety of gene editing by familiarity with gene editing.

The linear relationships between familiarity and strength of opinion are illustrated in Figure 1. Given the categorical response options for the two questions, Chi-square tests of independence were estimated for each sample and field to determine if opinion strength was independent of familiarity. The null hypotheses of independence were rejected for all tests. Thus, the strength of a respondent’s opinion about safety increased with familiarity with gene editing in the agricultural and medical fields in both samples.

**FIGURE 2:**
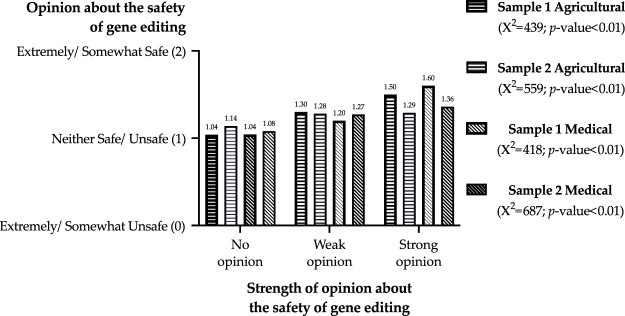
Opinion about the safety of gene editing by strength of opinion about safety.

Figure 2 illustrates the relationships between opinions about the safety of gene editing and opinion strength. While the null hypotheses of independence were also rejected by Chi-square tests estimated for these two variables, the linear relationships are much less pronounced than that of opinion strength and familiarity. Respondents with no opinion about safety were, on average, also likely to state an opinion of neither safe/unsafe. In contrast, respondents who did have an opinion about safety generally stated that the use of gene editing was safe. However, there were slight differences in opinions about safety between those with strong and weak opinions in sample 2; this is also highlighted by the correlations between the two variables, 0.08 for agriculture and 0.16 for medical (see Supplementary Table S2).

Ordered logistic regression models were estimated to determine the relative impact of familiarity and opinion strength on opinions about the safety of gene editing. The estimated coefficients are shown in Table 1. Familiarity was strongly associated with opinions about the safety of gene editing in the agricultural or medical fields; all coefficients were positive, indicating that the likelihood of agreeing that gene editing was safe increased with familiarity. After controlling for familiarity, the strength of an opinion about safety had little association with the opinion about the safety of gene editing. The strong opinion coefficient in the medical model for sample 1 was the only significant Strength of Opinion coefficient at a *p*-value less than 0.05, and it was positive, indicating that respondents with a strong opinion about the safety of gene editing were generally more agreeable that the use of gene editing in the medical field was safe (relative to respondents without an opinion).

**TABLE 1:** Ordered logistic regression coefficients for opinions about the safety of gene editing.

	Agricultural		Medical	
	Sample 1	Sample 2	Sample 1	Sample 2
Familiarity				
Yes, a little	0.915***	0.678***	1.043***	0.811***
	(0.142)	(0.089)	(0.142)	(0.086)
Yes, a lot	2.795***	1.505***	2.602***	1.783***
	(0.220)	(0.133)	(0.226)	(0.140)
Strength of Opinion				
Weak opinion	0.014	0.027	−0.269*	0.109
	(0.149)	(0.089)	(0.149)	(0.090)
Strong opinion	−0.033	−0.140	0.427**	0.127
	(0.180)	(0.106)	(0.189)	(0.110)
Log likelihood	−1,327	−3,200	−1,299	−3,098

Note: ***, **, and * denote *p*-value < 0.01, 0.05, and 0.10. Standard errors are reported in paratheses. There were 1,442 observations in the Sample 1 models and 3,125 observations in the Sample 2 models.

**FIGURE 3:**
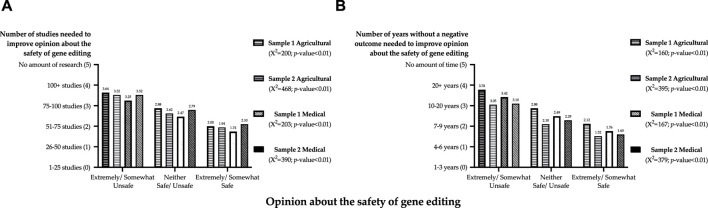
Amount of evidence necessary to improve opinions about the safety of gene editing by opinions about the safety of gene editing (Panel **(A)**: Number of studies; Panel **(B)**: Number of years).

Both samples required more evidence to improve opinions about the safety of gene editing in the medical field relative to agriculture. On average, respondents with a negative opinion required more than 100 studies or 10 years to improve opinions about the safety of gene editing. The relationships between the amount of evidence needed to improve opinions about the safety of gene editing and opinions about safety are shown in Figure 3. The number of studies needed to improve opinion is shown in Figure 3A; the amount of time without a negative consequence is shown in Figure 3B. The amount of evidence needed to improve opinions was not independent of opinions about safety; respondents who stated that gene editing was unsafe required more evidence to improve opinions about safety.

Ordered logistic regression models were estimated to determine the relative impact of familiarity, opinion strength, and opinion about safety on the amount of evidence needed to improve opinions. The estimated coefficients are shown in Table 2. The coefficients estimated for the Familiarity and Opinion about Safety variables were significant and negative in all models. These results indicate that respondents who were familiar with gene editing or did not hold a negative opinion about safety required less evidence to improve opinions about the safety of gene editing. The coefficients estimated for the Weak opinion variable were significant at a *p*-value less than 0.05 in all models for sample 1 and one model for sample 2, indicating that those with an opinion, but who have not formed a strong opinion, may be more amenable to evidence.

**TABLE 2:** Ordered logistic regression coefficients for evidence needed to improve opinions about the safety of gene editing.

	Agricultural				Medical			
Familiarity	Amount of Research		Amount of Time		Amount of Research		Amount of Time	
	Sample 1	Sample 2	Sample 1	Sample 2	Sample 1	Sample 2	Sample 1	Sample 2
Yes, a little	−0.710***	−0.340***	−0.510***	−0.277***	−0.611***	−0.376***	−0.376***	−0.322***
	(0.136)	(0.090)	(0.136)	(0.091)	(0.134)	(0.083)	(0.134)	(0.082)
Yes, a lot	−0.836***	−0.518***	−0.701***	−0.115	−0.831***	−0.524***	−0.486***	−0.277***
	(0.179)	(0.122)	(0.180)	(0.122)	(0.182)	(0.121)	(0.183)	(0.121)
Strength of Opinion								
Weak opinion	−0.416***	−0.141	−0.292**	−0.148*	−0.379***	−0.202**	−0.433***	0.043
	(0.141)	(0.086)	(0.142)	(0.087)	(0.139)	(0.085)	(0.139)	(0.084)
Strong opinion	−0.035	0.117	0.187	0.013	0.040	0.131	−0.030	0.129
	(0.163)	(0.101)	(0.162)	(0.102)	(0.165)	(0.102)	(0.165)	(0.102)
Opinion about Safety								
Neither Safe/Unsafe	−1.110***	−1.143***	−0.884***	−1.104***	−1.159***	−1.017***	−1.076***	−0.961***
	(0.149)	(0.097)	(0.146)	(0.095)	(0.146)	(0.098)	(0.142)	(0.095)
Extremely/Somewhat Safe	−1.605***	−1.740***	−1.374***	−1.680***	−1.652***	−1.605***	−1.608***	−1.670***
	(0.147)	(0.092)	(0.143)	(0.089)	(0.154)	(0.096)	(0.150)	(0.095)
Log likelihood	−2,434	−5,328	−2,440	−5,210	−2,428	−5,280	−2,442	−5,257

Note: ***, **, and * denote *p*-value < 0.01, 0.05, and 0.10. Standard errors are reported in paratheses. There were 1,442 observations in the Sample 1 models and 3,125 observations in the Sample 2 models.

## 4 Discussion

While the U.S. public is becoming more aware of gene editing in the medical field and may be more aware of the use of gene editing for medical purposes relative to transgenic approaches, results from this study indicate that the U.S. public may associate biotechnology more with agriculture. These findings are a valuable addition to existing literature that has examined perceptions of gene editing in agricultural or medical contexts separately. The prior qualitative study examining awareness about gene editing found uses in the medical field to be mentioned first in focus group discussions (McFadden et al., 2021a). The difference in results could be attributed to a few individuals in each group driving the conversation.

On average, respondents in both samples were more familiar with gene editing in agriculture, more likely to have an opinion about safety, have a more positive opinion, and require less evidence to improve opinions about safety than for medical purposes. The higher familiarity and opinion formation in the agricultural field may result from perception spillover from GMO food conversations. However, compared to the Pew Research study of GMOs, the slightly higher rates of perceived safety may indicate that negative perceptions toward safety do not spill over at a similar rate. This differs from the perception spillover observed in the context of energy innovation (Westlake et al., 2023), but could indicate that a larger percentage of our respondents perceive the safety of GMOs and gene editing in food as dissimilar. These nuances are essential to recognize as we continue engaging consumers in dialogue surrounding gene editing, ultimately impacting future innovation and policy. In particular, the findings suggest opportunities to share evidence of safety with those aware of the use of gene editing in agriculture. Doing so could generate more positive opinions, which individuals may share with others in their trusted social communities and create the opportunity for positive social influence.

Opinions about the safety of gene editing were generally more favorable for respondents who were aware or had formed an opinion about the safety of using gene editing within the agricultural or health field, supporting a proactive approach to messaging for gene editing and other technologies emerging into public consciousness. While familiarity and opinion strength were moderately correlated, familiarity was more closely associated with opinions about safety than opinion strength. The relationship between familiarity and opinions about safety may lead one to conclude that providing the public with more information about gene editing will improve opinions. However, the results of this study show that it may not be a valid conclusion for those with negative opinions about safety, and different approaches overall may be more meaningful for different public audiences. It also has been shown that scientific evidence is not compelling with some segments of the public and that strategies to build trust or rely on trusted messengers such as community leaders are more effective in changing perceptions (James, 2003). Together, these findings present the opportunity to explore strategies for designing tailored messaging and experiences around familiar gene-editing contexts within people’s values and belief systems and the potential for engagement to then lean on these community members’ positive attitudes to spread to others. Doing so could further enhance positive opinions toward and support for gene editing.

There are limitations to this study. One, no information was provided to respondents before the survey questions about gene editing in the agricultural and medical fields. Results are associated with the opinions that respondents attached to gene editing before participating in the survey, sometimes referred to as “homegrown” perceptions or values (Cummings et al., 1995; McFadden and Malone, 2021). The first question asked about familiarity, and thus, providing information could have primed some respondents. Future research could use a qualitative approach that would allow for follow-up discussions about the factors individuals affiliate with gene editing and what is considered in the scope of gene editing (e.g., transgenesis). Also, there are many nuances among gene editing applications, and familiarity with and opinions about genetic modification have been found to vary by the outcome of an application (Lusk et al., 2015) and even by the organization that developed the application (Lusk et al., 2018). Future research could focus on variations in familiarity with and opinions about gene editing, given specific application nuances. Lastly, there could have also been variations in how respondents interpreted research studies as evidence. For example, low-quality sources are more influential in forming opinions than more traditional sources (Aslett et al., 2023), and confirmation bias may result in individuals rejecting information from reputable sources if the information does not align with opinions before receiving the information (McFadden and Lusk, 2015).

## Data Availability

The datasets presented in this study can be found in online repositories. The names of the repository/repositories and accession number(s) can be found below: https://figshare.com/s/221190b7d727b759dac1.

## References

[B1] Aslett K. Sanderson Z. Godel W. Persily N. Nagler J. Tucker J. A. (2023). Online searches to evaluate misinformation can increase its perceived veracity. Nature 625, 548–556. 10.1038/s41586-023-06883-y 38123685 PMC10794132

[B2] Busch G. Ryan E. von Keyserlingk M. A. Weary D. M. (2022). Citizen views on genome editing: effects of species and purpose. Agric. Hum. Values 39 (1), 151–164. 10.1007/s10460-021-10235-9

[B3] Critchley C. Nicol D. Bruce G. Walshe J. Treleaven T. Tuch B. (2019). Predicting public attitudes toward gene editing of germlines: the impact of moral and hereditary concern in human and animal applications. Front. Genet. 9, 704. 10.3389/fgene.2018.00704 30687386 PMC6334182

[B4] Cummings R. G. Harrison G. W. Rutström E. E. (1995). Homegrown values and hypothetical surveys: is the dichotomous choice approach incentive-compatible? Am. Econ. Rev. 85 (1), 260–266.

[B5] Funk C. Rainie L. Page D. (2015). Public and scientists’ views on science and society. Washington, D.C., United States: Pew Research Center, 29.

[B6] Gaskell G. Bard I. Allansdottir A. Da Cunha R. V. Eduard P. Hampel J. (2017). Public views on gene editing and its uses. Nat. Biotechnol. 35 (11), 1021–1023. 10.1038/nbt.3958 29121022

[B7] Godlee F. (2010). More research is needed—but what type? BMJ 341, c4662. 10.1136/bmj.c4662

[B8] Hering J. G. (2016). Do we need “more research” or better implementation through knowledge brokering? Sustain. Sci. 11, 363–369. 10.1007/s11625-015-0314-8 30174733 PMC6106375

[B9] Holdren J. Shelanski H. Vetter D. Goldfuss C. (2019). Modernizing the regulatory system for biotechnology products. Washington, DC, USA: Office of Science and Technology Policy.

[B10] Holdren J. P. Sunstein C. R. Siddiqui I. A. (2011). Principles for regulation and oversight of emerging technologies. Washington, D.C., United States: Office of Science and Technology Policy.

[B11] James H. S. (2003). The effect of trust on public support for biotechnology: evidence from the US Biotechnology Study. Agribus. Int. J. 19 (2), 155–168. 10.1002/agr.10052

[B12] Jedwab A. Vears D. F. Tse C. Gyngell C. (2020). Genetics experience impacts attitudes towards germline gene editing: a survey of over 1500 members of the public. J. Hum. Genet. 65 (12), 1055–1065. 10.1038/s10038-020-0810-2 32737393

[B13] Kato-Nitta N. Maeda T. Inagaki Y. Tachikawa M. (2019). Expert and public perceptions of gene-edited crops: attitude changes in relation to scientific knowledge. Palgrave Commun. 5 (1), 137–144. 10.1057/s41599-019-0328-4

[B14] Kobayashi S. Miyoshi T. Kobayashi T. Hayakawa I. Urayama K. Y. Uchiyama M. (2022). Public attitudes in the clinical application of genome editing on human embryos in Japan: a cross-sectional survey across multiple stakeholders. J. Hum. Genet. 67, 541–546. 10.1038/s10038-022-01042-z 35534678

[B15] Liu H. Priest S. (2009). Understanding public support for stem cell research: media communication, interpersonal communication and trust in key actors. Public Underst. Sci. 18 (6), 704–718. 10.1177/0963662508097625

[B16] Lusk J. L. Jamal M. Kurlander L. Roucan M. Taulman L. (2005). A meta-analysis of genetically modified food valuation studies. J. Agric. Resour. Econ., 28–44. 10.22004/ag.econ.30782

[B17] Lusk J. L. McFadden B. R. Rickard B. J. (2015). Which biotech foods are most acceptable to the public? Biotechnol. J. 10 (1), 13–16. 10.1002/biot.201400561 25388815

[B18] Lusk J. L. McFadden B. R. Wilson N. (2018). Do consumers care how a genetically engineered food was created or who created it? Food Policy 78, 81–90. 10.1016/j.foodpol.2018.02.007

[B19] McCaughey T. Budden D. M. Sanfilippo P. G. Gooden G. E. Fan L. Fenwick E. (2019). A need for better understanding is the major determinant for public perceptions of human gene editing. Hum. gene Ther. 30 (1), 36–43. 10.1089/hum.2018.033 29926763

[B20] McFadden B. R. Anderton B. N. Davidson K. A. Bernard J. C. (2021b). The effect of scientific information and narrative on preferences for possible gene-edited solutions for citrus greening. Appl. Econ. Perspect. Policy 43 (4), 1595–1620. 10.1002/aepp.13154

[B21] McFadden B. R. Lusk J. L. (2015). Cognitive biases in the assimilation of scientific information on global warming and genetically modified food. Food Policy 54, 35–43. 10.1016/j.foodpol.2015.04.010

[B22] McFadden B. R. Malone T. (2021). Homegrown perceptions about the medical use and potential abuse of CBD and THC. Addict. Behav. 115, 106799. 10.1016/j.addbeh.2020.106799 33387977

[B23] McFadden B. R. Rumble J. N. Stofer K. A. Folta K. M. Turner S. Pollack A. (2021a). Gene editing isn’t just about food: comments from US focus groups. GM Crops Food. 12 (2), 616–626. 10.1080/21645698.2021.1919485 34014805 PMC9208619

[B24] National Academies of Sciences (2016). “Engineering, and medicine,” in Genetically engineered crops: experiences and prospects (Washington, D.C., United States: National Academies Press). 28230933

[B25] National Academies of Sciences (2017). “Engineering, and medicine (NASEM),” in Human genome editing: science, ethics, and governance (Washington, D.C., United States: National Academies Press). 28796468

[B26] Scott S. E. Inbar Y. Rozin P. (2016). Evidence for absolute moral opposition to genetically modified food in the United States. Perspect. Psychol. Sci. 11 (3), 315–324. 10.1177/1745691615621275 27217243

[B27] Simis M. J. Madden H. Cacciatore M. A. Yeo S. K. (2016). The lure of rationality: why does the deficit model persist in science communication? Public Underst. Sci. 25 (4), 400–414. 10.1177/0963662516629749 27117768

[B28] Treleaven T. Tuch B. E. (2018). Australian public attitudes on gene editing of the human embryo. J. Law Med. 26, 204–207. 30302982

[B29] Watanabe D. Saito Y. Tsuda M. Ohsawa R. (2020). Increased awareness and decreased acceptance of genome-editing technology: the impact of the Chinese twin babies. PloS one 15 (9), e0238128. 10.1371/journal.pone.0238128 32946484 PMC7500613

[B30] Westlake S. John C. H. D. Cox E. (2023). Perception spillover from fracking onto public perceptions of novel energy technologies. Nat. Entergy 8, 149–158. 10.1038/s41560-022-01178-4

[B31] Yang Y. Hobbs J. E. (2020). Supporters or opponents: will cultural values shape consumer acceptance of gene editing? J. Food Prod. Mark. 26 (1), 17–37. 10.1080/10454446.2020.1715316

[B32] Zhang X. Chen A. Zhang W. (2021). Before and after the Chinese gene-edited human babies: multiple discourses of gene editing on social media. Public Underst. Sci. 30 (5), 570–587. 10.1177/0963662520987754 33467986

