# Supplementary material for: U.S. public opinion about the safety of gene editing in the agriculture and medical fields and the amount of evidence needed to improve opinions
Source: Front Bioeng Biotechnol. 2024 Feb 16;12:1340398. doi: 10.3389/fbioe.2024.1340398 (PMC10904643; doi:10.3389/fbioe.2024.1340398)
Supplement: Supplementary file 1 [file DataSheet1.docx]

Supplementary Information


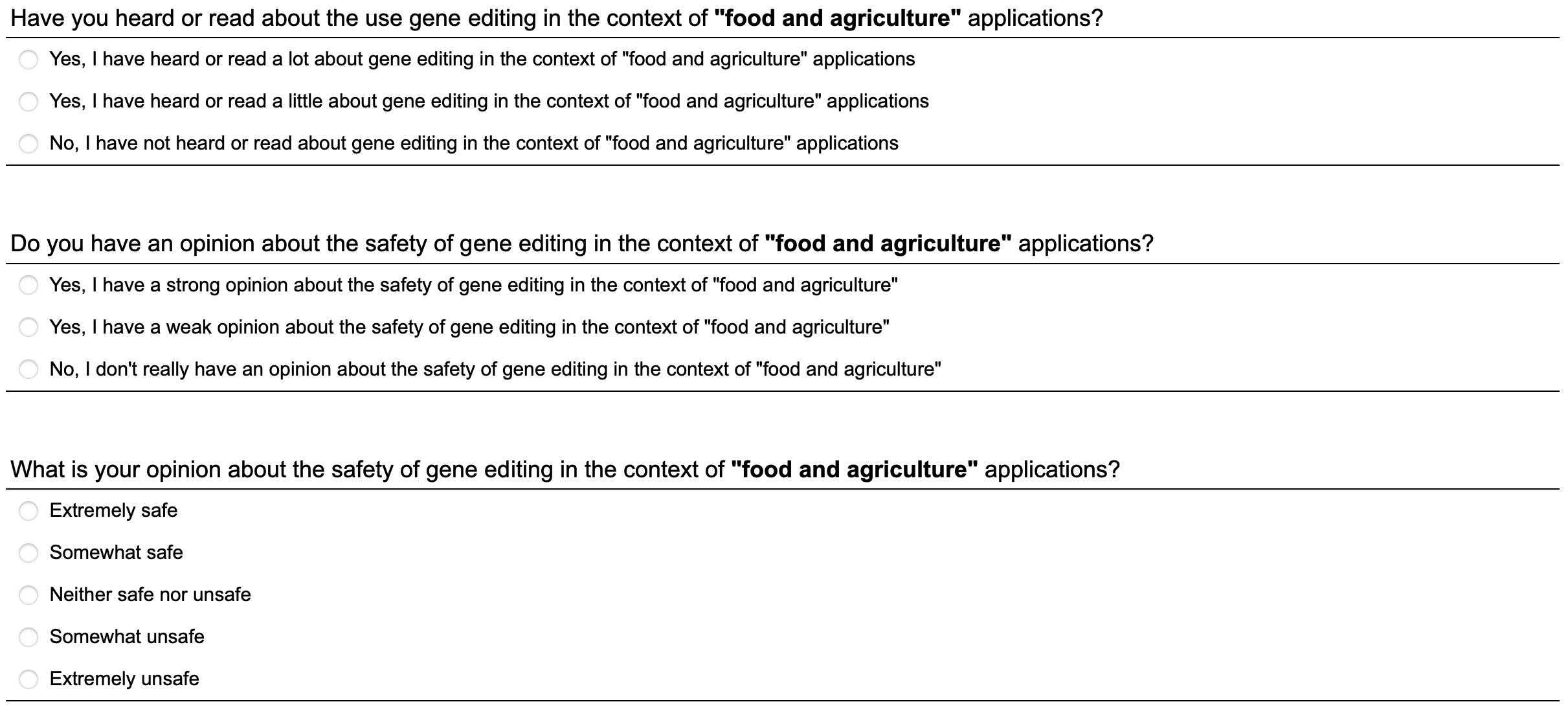


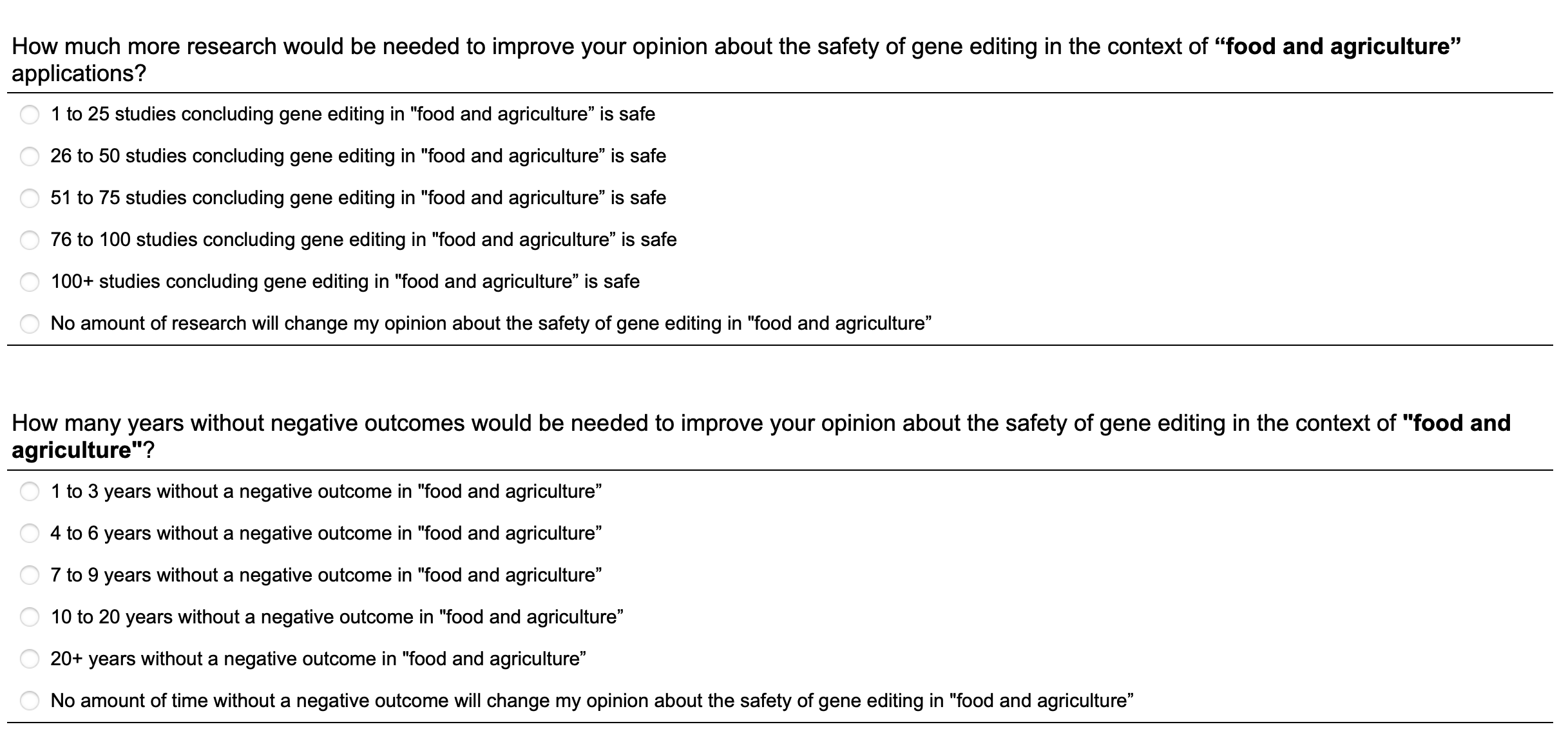


Supplementary Information Figure 1. Survey questions for gene editing in food and agriculture


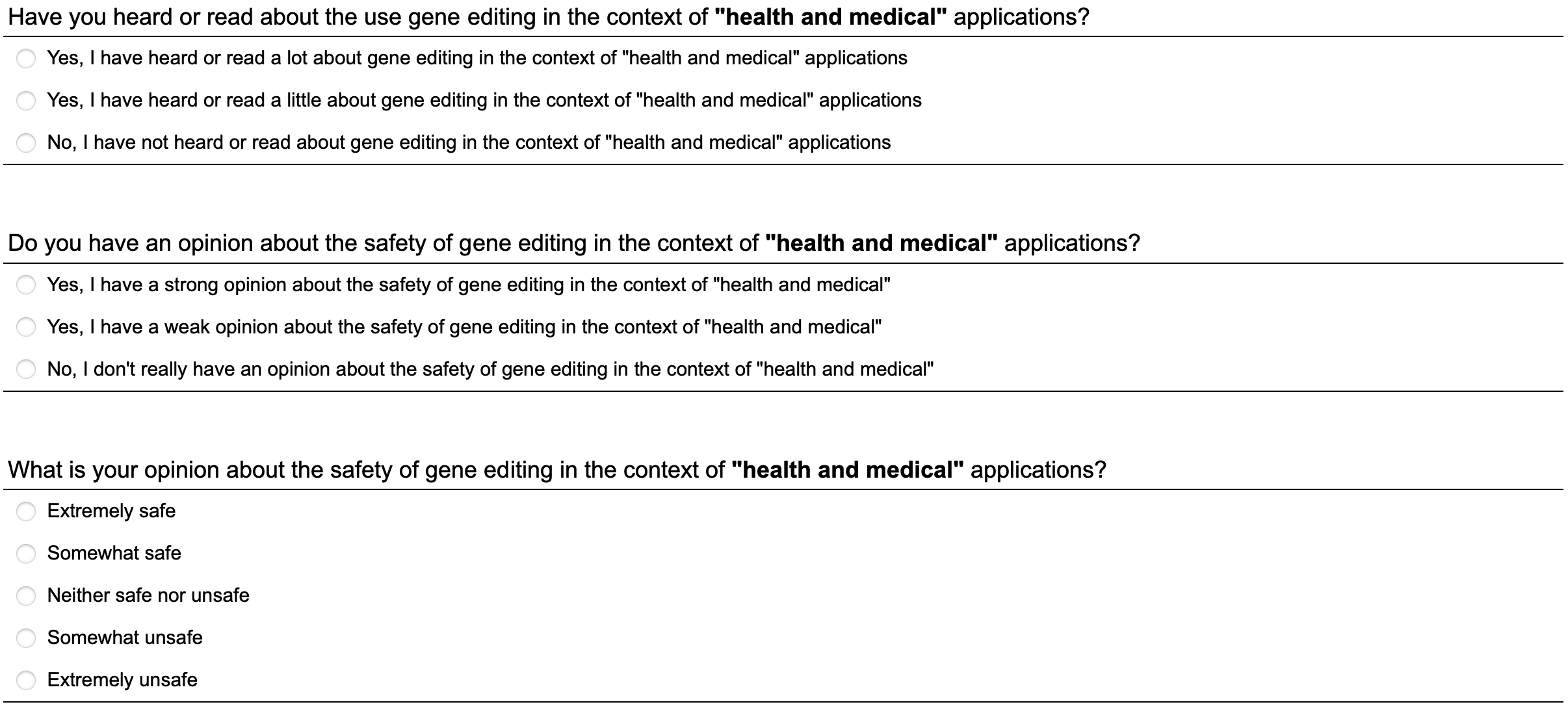


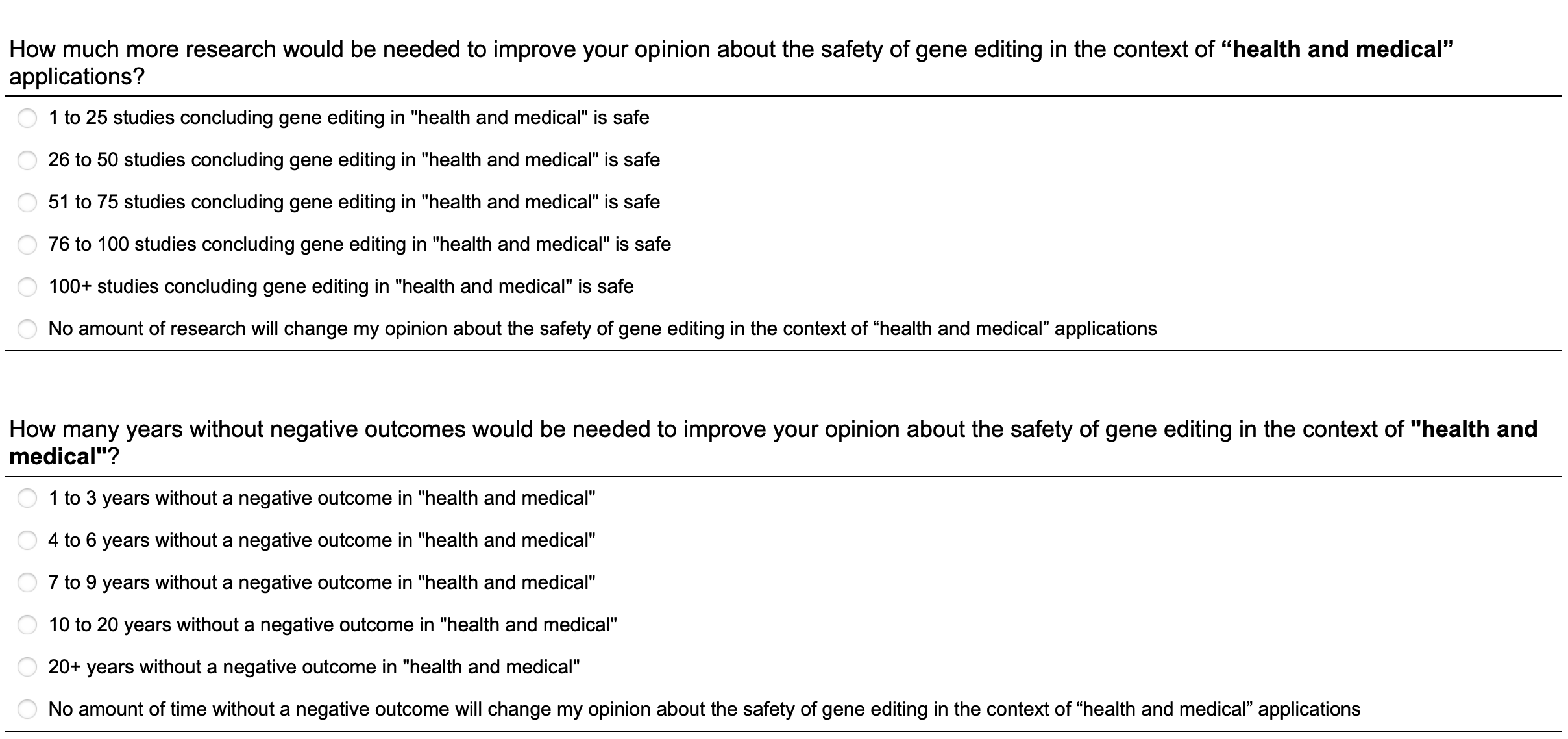


Supplementary Information Figure 2. Survey questions for gene editing in health and medical


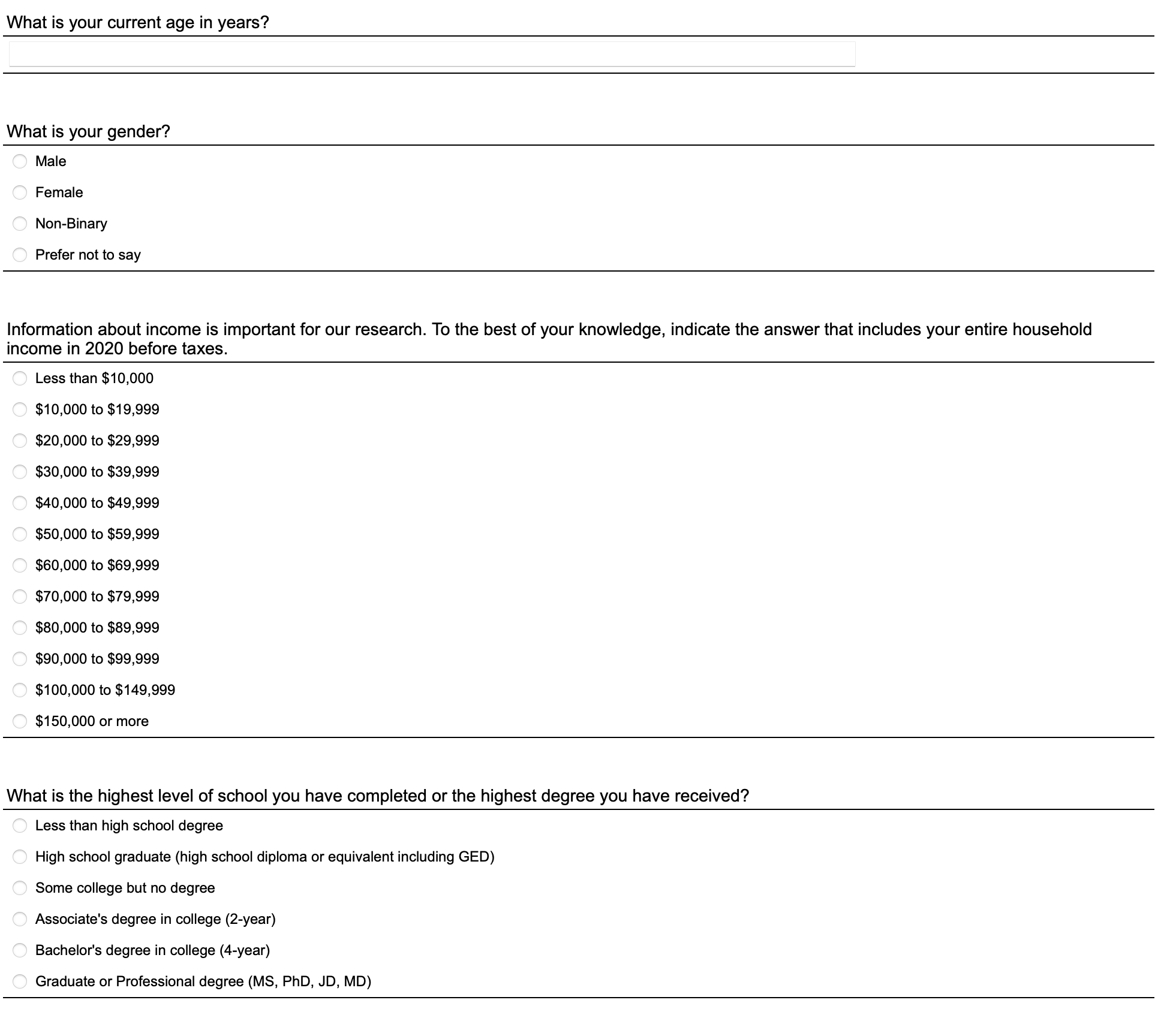


Supplementary Information Figure 3. Survey questions for the characteristics of respondents


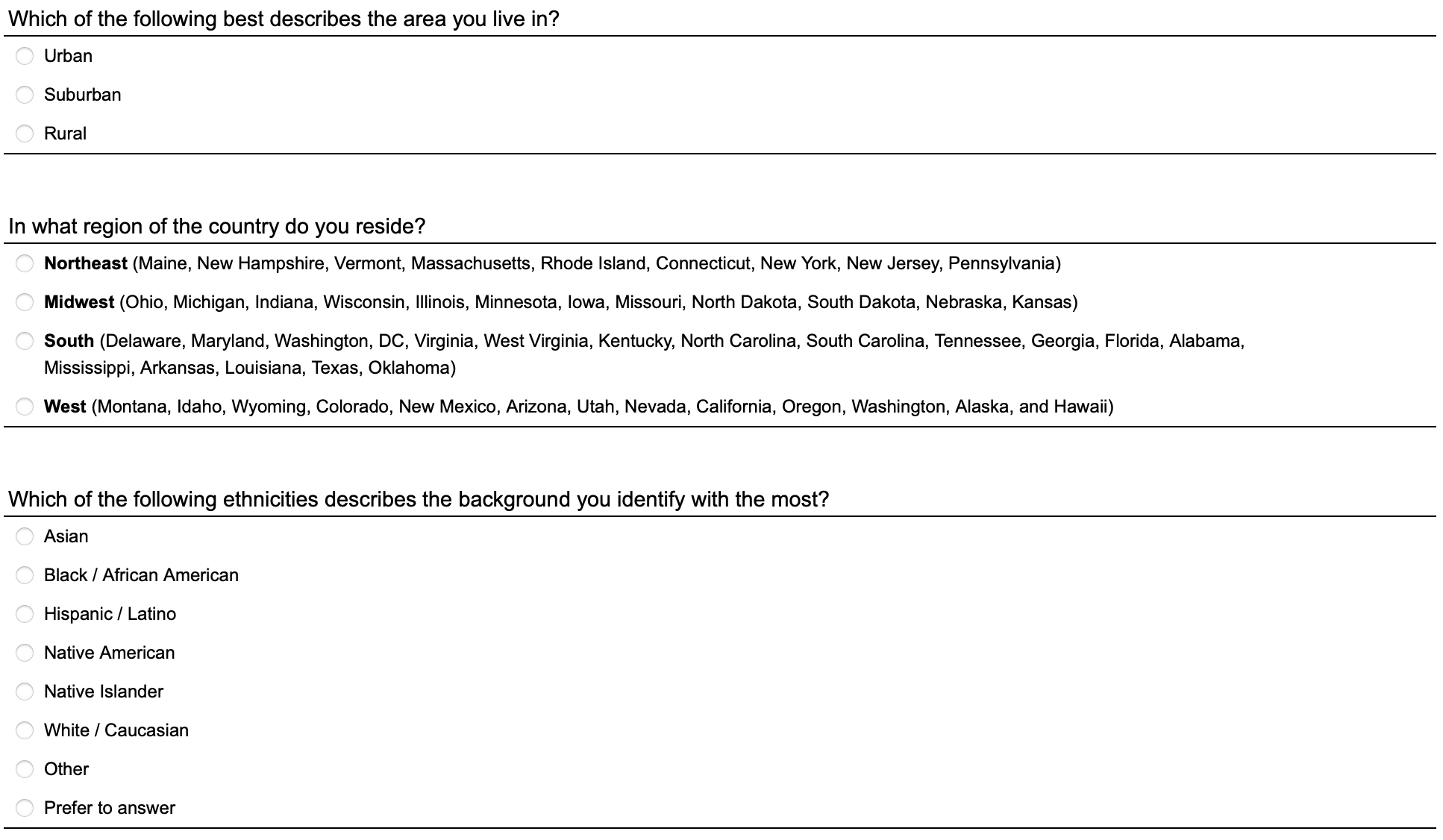


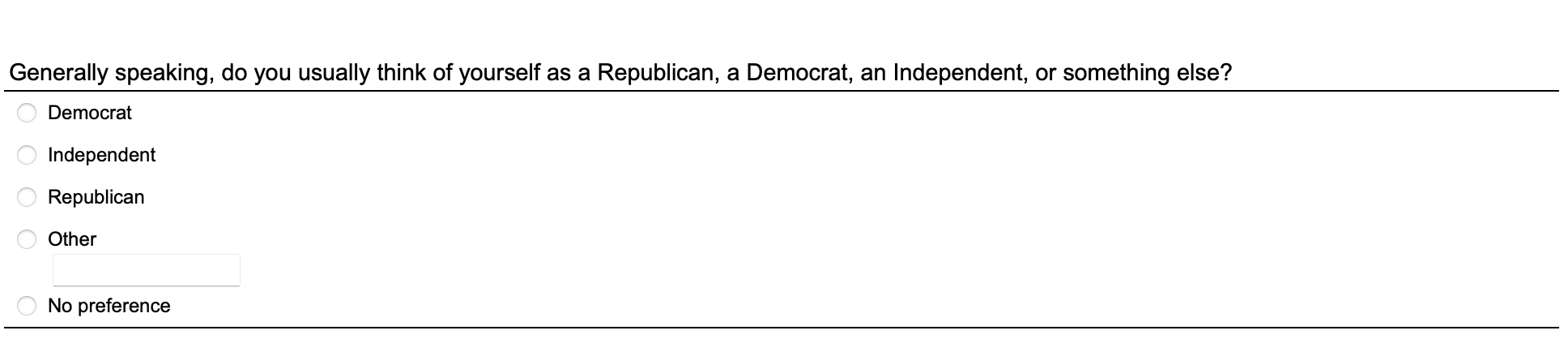


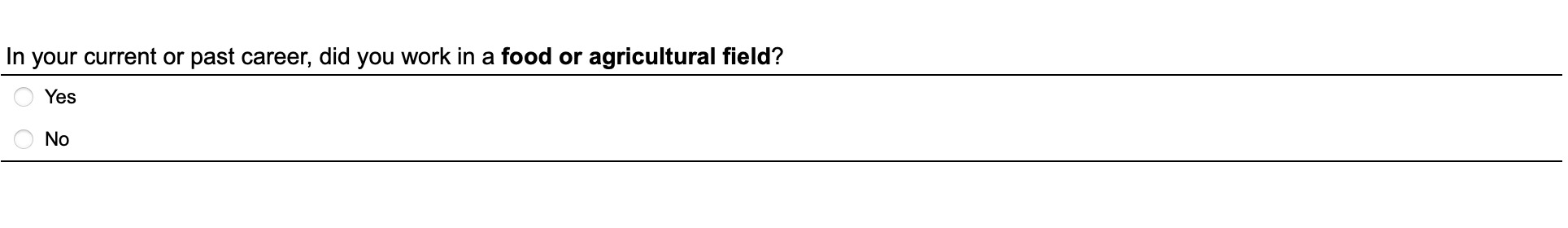


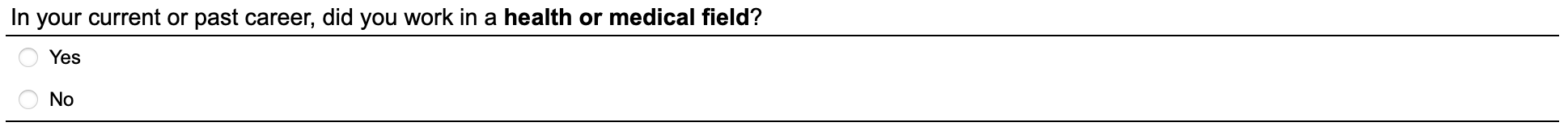


Supplementary Information Figure 3 (continued). Survey questions for the characteristics of respondents

| Supplementary Information Table 1. Frequency distributions (%) for the characteristics of respondents | | |
| --- | --- | --- |
| Respondents’ characteristics | Sample 1 (n=1,442) | Sample 2 (n=3,125) |
| **Age**^1^ | 43.55 | 44.39 |
| **Gender** |  |  |
| Female | 51.32 | 54.82 |
| Male | 46.60 | 43.78 |
| Non-binary | 1.73 | 1.22 |
| Prefer not to say | 0.35 | 0.19 |
| **Income** |  |  |
| Less than $10,000 | 7.70 | 6.94 |
| $10,000 to $19,999 | 9.36 | 8.32 |
| $20,000 to $29,999 | 11.23 | 11.33 |
| $30,000 to $39,999 | 10.61 | 10.62 |
| $40,000 to $49,999 | 7.98 | 8.42 |
| $50,000 to $59,999 | 7.77 | 10.72 |
| $60,000 to $69,999 | 4.30 | 6.98 |
| $70,000 to $79,999 | 6.31 | 8.77 |
| $80,000 to $89,999 | 2.36 | 4.67 |
| $90,000 to $99,999 | 4.44 | 5.57 |
| $100,000 to $149,999 | 16.57 | 12.42 |
| $150,000 or more | 11.37 | 5.25 |
| **Education** |  |  |
| Less than high school degree | 4.85 | 3.78 |
| High school graduate | 26.56 | 25.18 |
| Some college but no degree | 30.37 | 28.54 |
| Associate degree in college | 5.27 | 11.01 |
| Bachelor’s degree in college | 15.81 | 20.42 |
| Graduate or Professional degree | 17.13 | 11.07 |
| **Density** |  |  |
| Rural | 21.98 | 22.82 |
| Suburban | 45.98 | 50.24 |
| Urban | 32.04 | 26.94 |
| **Census Region** |  |  |
| Midwest | 24.55 | 21.37 |
| Northeast | 19.76 | 18.40 |
| South | 36.06 | 42.85 |
| West | 19.63 | 17.38 |
| **Ethnicity / Race** |  |  |
| Asian | 5.55 | 4.00 |
| Black / African American | 8.04 | 14.27 |
| Hispanic / Latino | 5.34 | 7.01 |
| Native American | 1.39 | 1.15 |
| Native Islander | 0.28 | 0.26 |
| White / Caucasian | 77.25 | 71.26 |
| Other | 1.39 | 1.47 |
| Prefer to answer | 0.76 | 0.58 |
| **Political Affiliation** |  |  |
| Democrat | 40.15 | 36.03 |
| Republican | 22.75 | 27.58 |
| Independent / Other | 37.10 | 36.39 |
| **Career Experience** |  |  |
| Agricultural / Food | 18.45 | 19.49 |
| Health / Medical | 18.10 | 13.98 |
| ^1^ Note: The means are reported for age, not frequency distributions, because age was measured as a continuous variable. | | |

| Supplementary Table 2. Frequency distributions (%) for variables and differences in responses between Agricultural and Medical Applications | | | | | |
| --- | --- | --- | --- | --- | --- |
|  | Sample 1 (n=1,442) | |  | Sample 2 (n=3,125) | |
|  | *Agricultural* | *Medical* |  | *Agricultural* | *Medical* |
| **Familiarity** | z = 4.6; *p*-value < 0.01 | |  | z = 14.0; *p*-value < 0.01 | |
| No (0) | 34.12 | 39.39 |  | 24.99 | 36.83 |
| Yes, I have heard or read a little (1) | 42.16 | 38.56 |  | 56.86 | 48.83 |
| Yes, I have heard or read a lot (2) | 23.72 | 22.05 |  | 18.14 | 14.34 |
|  |  |  |  |  |  |
| **Strength of opinion about safety** | z = 4.0; *p*-value < 0.01 | |  | z = 9.4; *p*-value < 0.01 | |
| No (0) | 38.07 | 43.55 |  | 33.63 | 41.54 |
| Yes, I have a weak opinion (1) | 31.41 | 28.36 |  | 37.41 | 34.27 |
| Yes, I have a strong opinion (2) | 30.51 | 28.09 |  | 28.96 | 24.19 |
|  |  |  |  |  |  |
| **Opinion about safety** | z = 1.1; *p*-value = 0.29 | |  | z = 1.3; *p*-value = 0.21 | |
| Extremely/Somewhat Unsafe (0) | 17.75 | 16.78 |  | 20.54 | 18.30 |
| Neither Safe/Unsafe (1) | 38.49 | 42.09 |  | 35.42 | 41.95 |
| Extremely/Somewhat Safe (2) | 43.76 | 41.12 |  | 44.03 | 39.74 |
|  |  |  |  |  |  |
| **Amount of research** | z = -4.0; *p*-value < 0.01 | |  | z = -6.6; *p*-value < 0.01 | |
| 1-25 studies (0) | 14.70 | 13.38 |  | 15.20 | 12.48 |
| 26-50 studies (1) | 18.10 | 16.71 |  | 18.85 | 17.92 |
| 51-75 studies (2) | 15.81 | 16.23 |  | 16.77 | 15.65 |
| 76-100 studies (3) | 12.62 | 11.93 |  | 13.02 | 13.66 |
| 100+ studies (4) | 18.45 | 21.84 |  | 21.41 | 26.88 |
| No amount of research will improve opinion (5) | 20.32 | 19.90 |  | 14.75 | 13.41 |
|  |  |  |  |  |  |
| **Amount of time** | z = -3.3; *p*-value < 0.01 | |  | z = -7.2; *p*-value < 0.01 | |
| 1-3 years (0) | 20.11 | 17.61 |  | 20.35 | 16.54 |
| 4-6 years (1) | 21.57 | 22.95 |  | 26.21 | 25.18 |
| 7-9 years (2) | 14.77 | 14.49 |  | 15.87 | 16.70 |
| 10-20 (3) | 15.60 | 15.81 |  | 16.83 | 20.13 |
| 20+ years (4) | 8.74 | 10.40 |  | 8.26 | 9.47 |
| No amount of time without a negative outcome will improve opinion (5) | 19.21 | 18.72 |  | 12.48 | 11.97 |
| Note: The z-scores and associated *p*-values were estimated using Wilcoxon matched-pairs signed-rank tests to determine differences in responses between Agricultural and Medical. | | | | | |

| Supplementary Information Table 3. Correlations between responses for agricultural or medical gene-edited applications | | | | | | | | | | | | | | | | | | | | | | | |
| --- | --- | --- | --- | --- | --- | --- | --- | --- | --- | --- | --- | --- | --- | --- | --- | --- | --- | --- | --- | --- | --- | --- | --- |
| Agricultural | | | | | | | | | | | | | | | | | | | | | | | |
| *Sample 1* | | | | | | | | | | | | *Sample 2* | | | | | | | | | | | |
|  | | Familiarity | | Strength  of Opinion | | Opinion about Safety | | Amount of Research | | Amount of Time | |  | | Familiarity | | | Strength of Opinion | | Opinion about Safety | | Amount of Research | | Amount of Time |
| Familiarity | | 1 | |  | |  | |  | |  | | Familiarity | | 1 | | |  | |  | |  | |  |
| Strength of Opinion | | 0.67 | | 1 | |  | |  | |  | | Strength of Opinion | | 0.56 | | | 1 | |  | |  | |  |
| Opinion about Safety | | 0.41 | | 0.26 | | 1 | |  | |  | | Opinion about Safety | | 0.21 | | | 0.08 | | 1 | |  | |  |
| Amount of Research | | -0.27 | | -0.17 | | -0.35 | | 1 | |  | | Amount of Research | | -0.13 | | | -0.03 | | -0.35 | | 1 | |  |
| Amount of Time | | -0.21 | | -0.10 | | -0.31 | | 0.66 | | 1 | | Amount of Time | | -0.08 | | | -0.02 | | -0.35 | | 0.60 | | 1 |
|  |  | |  | |  | |  | |  | |  | |  | |  |  | |  | |  | |  | |
| Medical | | | | | | | | | | | | | | | | | | | | | | | |
| *Sample 1* | | | | | | | | | | | | *Sample 2* | | | | | | | | | | | |
|  | | Familiarity | | Strength of Opinion | | Opinion about Safety | | Amount of Research | | Amount of Time | |  | | Familiarity | | | Strength of Opinion | | Opinion about Safety | | Amount of Research | | Amount of Time |
| Familiarity | | 1 | |  | |  | |  | |  | | Familiarity | | 1 | | |  | |  | |  | |  |
| Strength of Opinion | | 0.70 | | 1 | |  | |  | |  | | Strength of Opinion | | 0.56 | | | 1 | |  | |  | |  |
| Opinion about Safety | | 0.43 | | 0.32 | | 1 | |  | |  | | Opinion about Safety | | 0.28 | | | 0.16 | | 1 | |  | |  |
| Amount of Research | | -0.27 | | -0.18 | | -0.34 | | 1 | |  | | Amount of Research | | -0.16 | | | -0.07 | | -0.32 | | 1 | |  |
| Amount of Time | | -0.23 | | -0.16 | | -0.33 | | 0.63 | | 1 | | Amount of Time | | -0.14 | | | -0.05 | | -0.34 | | 0.58 | | 1 |
